# The co-delivery of Programmed Death 1 ligands enhances and prolongs rAAV-mediated gene expression in pre-immunized mice

**DOI:** 10.1038/s41434-025-00588-9

**Published:** 2026-01-09

**Authors:** Piia Käyhty, Tiina Nieminen, Reetta A. E. Eriksson, Ritva Tumelius, Ahmed Tawfek, Svetlana Laidinen, Anna-Kaisa Ruotsalainen, Aubrey Bailey, Lionel Galibert, Hanna P. Lesch, Seppo Ylä-Herttuala, Kari J. Airenne

**Affiliations:** 1 Ferring Ventures Oy, Kuopio, Finland; 2 A.I.Virtanen Institute for Molecular Sciences, University of Eastern Finland, Kuopio, Finland; 3 Heart Center, Kuopio University Hospital, Kuopio, Finland; 4 Gene Therapy Unit, Kuopio University Hospital, Kuopio, Finland

**Keywords:** Gene therapy, Immunotherapy

## Abstract

Immune responses against recombinant adeno-associated virus (rAAV) are one of the major obstacles in gene therapy. We investigated the potential of Programmed Death 1 ligands 1 and 2 (PD-L1/2) to protect AAV-transduced cells from immunological clearance. Ligand compatibility for co-delivery was first evaluated using two transgenes, *VEGF-B186* and *muSEAP*, separated from PD-L1/2 by a self-cleaving P2A peptide. After proper cleavage and biological activity of the co-produced proteins were demonstrated in vitro, the effect of PD-L1/2 co-expression on muSEAP production and persistence was studied in naïve and vector pre-immunized mice. Vectors (rAAV6-muSEAP, rAAV6-muSEAP-PD-L1, or rAAV6-muSEAP-PD-L2) were injected into two sites of the gastrocnemius muscle at a total dose of 1×10^10^ vg. Co-delivery of PD-L1, particularly, significantly enhanced muSEAP secretion into the bloodstream up to 12 weeks despite elevated anti-AAV6 responses in pre-immunized mice. muSEAP secretion increased 33.3- and 31.4-fold with the co-delivery of PD-L1, while the increase was only 5.6- and 9.3-fold in the muSEAP control group at 5 and 12 weeks, respectively. Ligand-treated pre-immunized animals also had less T-cell infiltration into the treated muscle compared to naïve animals. In summary, co-delivery of PD-L1/2 alongside a transgene represents a promising strategy for achieving sustained gene expression in individuals pre-exposed to AAV.

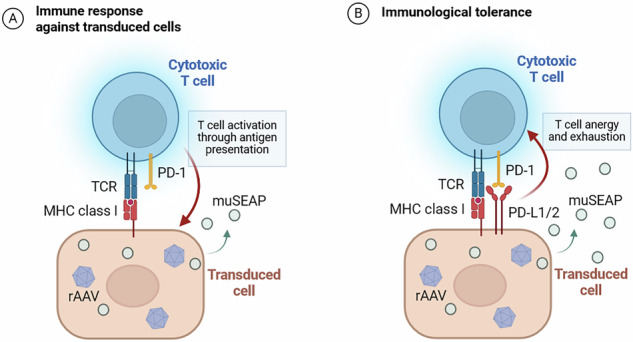

## Introduction

Adeno-associated virus (AAV) is a small dependoparvovirus with a single-stranded DNA genome. As a gene therapy vector, it can support long-lasting gene expression and has a broad, natural serotype-dependent or engineered tissue tropism. Recombinant AAVs (rAAVs) are widely used to treat ocular [1], neurological [2], neuromuscular [3–5], and other genetic disorders [6–9]. Although rAAVs are considered safe and non-pathogenic [10], prior exposure to wild-type AAV infections in humans triggers serotype cross-reactive immune responses, which limit the clinical applications of rAAV-based gene therapies [11, 12]. Immune responses against capsid-derived antigens can lead to reduced transgene expression and limit sustained therapeutic benefit, particularly when transduced cells are targeted by cytotoxic T cells [3, 8, 12–15]. Additionally, humans may harbor serotype-cross-reactive memory CD8^+^ T cells that become reactivated during rAAV-mediated gene transfer [12]. Furthermore, certain therapeutic proteins or their overexpression can also provoke immune responses [14, 16–18].

Skeletal muscle is a key target for gene therapy in metabolic [6, 19], ischemic [20, 21], and neuromuscular [3, 4, 17] diseases. Intramuscular (IM) gene transfer requires careful balancing of pro- and anti-inflammatory responses to the vector and the transgene. Disease-associated inflammation can increase transgene immunogenicity [17], although in some cases, IM gene transfer may promote a tolerogenic environment by promoting the activation and accumulation of regulatory T cells (Tregs) [22]. Current strategies to mitigate AAV immunogenicity include excluding seropositive patients or administering immunosuppressive agents such as corticosteroids, calcineurin inhibitors, or tacrolimus [23]. Additional approaches, such as capsid engineering, plasmapheresis, rapamycin nanoparticles, and Toll-like receptor 9 inhibitors, have been explored in preclinical studies [23–25]. However, these methods carry risks, including increased susceptibility to infections and malignancies.

One potential solution to the challenge of AAV immunogenicity is the induction of peripheral tolerance, which could suppress immune activation and thereby enhance both the efficacy and longevity of transgene expression. This may be achieved by taking advantage of the immune checkpoint Programmed Death 1 (PD-1) pathway. PD-1 binding to its ligands, PD-L1 and PD-L2, regulates T-cell signaling and plays a key role in maintaining natural self-tolerance and preventing autoimmune reactions [26, 27]. Overexpression of PD-L1 has previously been shown to protect transduced tissues and organs in transplantation models. For example, Yoshihara et al. [28] used a lentiviral vector to transduce pluripotent stem cells, which were subsequently differentiated into islet-like organoids, to protect the transplanted cells from autoimmune β-cell destruction.

In this study, we evaluated the feasibility of PD-L1/2 co-expression with a therapeutic transgene and assessed its potential to enhance gene expression in mice. We first investigated *murine secreted embryonic alkaline phosphatase* (*muSEAP*) or *vascular endothelial growth factor B* (*VEGF-B*), paired with *PD-L1* or *PD-L2*, and tested their compatibility in vitro using plasmid transfections to optimize P2A orientation in the expression cassette for efficient cleavage and functional protein production. Next, we investigated the effect of this co-delivery approach on rAAV-mediated gene delivery in the skeletal muscle of naïve and vector pre-immunized mice (PI). Our findings suggest that the co-delivery strategy is a viable and promising approach to improve sustained gene expression in AAV pre-exposed individuals.

## Materials and methods

### Plasmids and viral vectors

PD-L1-P2A-VEGF-B186 and VEGF-B186-P2A-PD-L1 constructs, with and without codon optimization, were synthesized by GenScript (Piscataway, NJ, USA). Coding DNA sequences were optimized using the GeneArt algorithm (Thermo Fisher Scientific, Waltham, MA, USA). Constructs were further subcloned into the pAdApt expression vector (Thermo Fisher Scientific) for characterization. For subsequent cloning into the pAAV-ITR backbone, muSEAP-P2A-PD-L1, muSEAP-P2A-PD-L2, VEGF-B186-P2A-PD-L1, and VEGF-B186-P2A-PD-L2 were synthetized (GeneWiz, South Plainfield, NJ, USA) in pUC57 backbone surrounded by SpeI and HpaI restriction sites. The muSEAP coding sequence was not codon-optimized; however, PD-L1 and PD-L2 sequences in muSEAP constructs were identical to the optimized sequences in other constructs. muSEAP and VEGF-B186 plasmids without ligands, and the vector production was described by Eriksson et al. [29]. All constructs were under a CMV promoter. The schematic presentation of each construct is shown in Supplementary Fig. 1.

### Transfection and transduction assays

293T cells (ECACC 12022001, RRID: CVCL_0063) were transfected with polyethylenimine (PEIpro, Polyplus transfection, Illkirch-Graffenstaden, France) and expression plasmids at a 1:1 ratio to produce PD-L1, PD-L2, VEGF-B186, and muSEAP proteins. Cell culture supernatants were collected at 48 h post-transfection, and cells were lysed with RIPA lysis and extraction buffer (Thermo Fisher Scientific) or NP-40 buffer (150 mM NaCl, 1% NP-40, 50 mM Tris-HCl, pH 8.0). Both lysis buffers were supplied with protease inhibitor cocktail (Hoffmann-La Roche, Basel, Switzerland).

rAAV6 transductions were carried out at a multiplicity of infection of 20000. An hour before transductions, cells were treated with 5 µM Dorsomorphin (Sigma-Aldrich, Saint Louis, MO, USA) to enhance transduction efficacy. Cell culture supernatants and cell lysates were collected at 72 h post-transduction.

### Immunoblotting

PD-L1, PD-L2, VEGF-B186, and muSEAP proteins were analyzed in 293T cell RIPA lysates and culture media using immunoblotting. Primary antibodies used were human PD-L1 antibody (cat# 13684, Cell Signaling Technologies, Danvers, MA, USA), human PD-L2 antibody (cat# 82723, Cell Signaling Technologies), human VEGF-B167/186 antibody (cat# AF751, R&D Systems, Minneapolis, MN, USA), human alkaline phosphatase antibody (cat# sc-398461, Santa Cruz Biotechnology, Dallas, TX, USA), and GAPDH (cat# 2118, Cell Signaling Technologies). Secondary antibodies used were Goat anti-Rabbit HRP (Bio Rad, Hercules, CA, USA, and Invitrogen, Waltham, Massachusetts, USA), Donkey anti-Goat HRP (R&D Systems), and Goat anti-Mouse HRP (Bio Rad). Restore™ PLUS Western Blot Stripping Buffer (Thermo Fisher Scientific) was used to remove bound primary and secondary antibodies from membranes before reprobing with another primary antibody.

### Cell growth/survival assay

BaF3-R1 cells [30], received as a generous gift from Dr. Kari Alitalo, were used to analyze the biological activity of VEGF-B186. Cells, normally dependent on recombinant mouse interleukin-3 for growth and survival, were incubated with media from VEGF-B186-transfected 293T cells under recombinant mouse interleukin-3 free conditions for 48 h. Cell Titer 96 Aqueous One Solution Cell Proliferation Assay reagent (Promega, Madison, WI, USA) was added to each well and incubated for two more hours. Absorbance was measured at 490 and 700 nm using Varioskan Lux with SkanIt Software. The 700 nm values were subtracted from the 490 nm values to reduce the possible background signal.

### SEAP reporter gene assay

A Phospha-Light^TM^ System chemiluminescence assay (Applied Biosystems, Foster City, CA, USA) was used to measure muSEAP protein in transfected or transduced 293T cell media and mouse sera. The assay was conducted according to the manufacturer’s instructions.

### Pulldown assay with recombinant PD-1-His

The binding of PD-L1/2 to PD-1 was analyzed using a pulldown assay. NP-40 lysates of transfected 293T cells were precleared with HisPur Ni-NTA Magnetic Beads (Thermo Scientific). Lysates were collected, supplied with 2.5 µg of PD1-His (cat# 8986-PD-100, R&D Systems) recombinant protein, and incubated at 4 °C overnight. Controls were incubated without PD1-His. The next day, the protein complexes were precipitated using HisPur Ni-NTA Magnetic Beads. Beads were washed with TBS containing 0.1% Tween, and protein complexes were eluted with PBS containing 500 mM imidazole (Sigma-Aldrich) at pH 8.0. Eluates were analyzed by immunoblotting with human PD-L1 and PD-L2 antibodies. Non-specific binding was controlled using the same blots and detection with a human Alkaline Phosphatase antibody and a human VEGF-B167/186 antibody. In addition, a human PD-1 antibody (cat# AMAb91197, Atlas Antibodies, Bromma, Sweden) was used to monitor recombinant PD-1 protein levels in samples.

### Mice

A total of seventy 9- or 10-week-old female C57BL/6JOlaHsd mice were obtained and kept in the National Laboratory Animal Center of The University of Eastern Finland, Kuopio, Finland. No sample size calculation was performed for the experiments. Animal work was conducted according to the animal experimentation license approved by the National Animal Experiment Board of the Regional State Administrative Agency of Southern Finland and carried out following the guidelines of the Finnish Act on Animal Experimentation (62/2006).

Half of the mice (*n* = 36) were pre-immunized with rAAV6-LacZ by IM injection into the left gastrocnemius medialis two weeks prior to gene transfer. For injection, mice were anesthetized with isoflurane, and 5×10^9^ vg in 12.5 µl was injected. For gene transfer, two weeks after pre-immunization, rAAV6-muSEAP-PD-L1 (*n* = 12), rAAV6-muSEAP-PD-L2 (*n* = 10), or rAAV6-muSEAP (*n* = 10) vectors were injected at 5 × 10^9^ vg in 12.5 µl into the right gastrocnemius medialis and gastrocnemius lateralis, with one dose into each muscle. The negative control mice (*n* = 4) received an equal volume of PBS. The animals were assigned to different groups without randomization. Blood samples were collected from the vena saphena prior to pre-immunization and every other week after gene transfer, up to 5 or 12 weeks after gene transfer. All experiments were performed unblinded.

### RNA isolation and RT-qPCR

RT-qPCR was used to quantify transgene products from mouse tissues. After sacrification, tissues were collected in liquid nitrogen and stored at –80 °C. Total RNA from approximately 50–100 mg of treated gastrocnemius muscle and liver was extracted using TRI Reagent (Invitrogen) according to the manufacturer’s instructions. QuantiTect Reverse Transcription kit (Qiagen, Hilden, Germany) was used to transcribe 1 µg of RNA into cDNA. Quantitative measurements of mRNA levels were performed using PowerUp SYBR Green Master Mix (Thermo Fisher Scientific) and 10 pmol of primers (Supplementary Table 1) with the StepOnePlus Real-Time PCR System (Thermo Fisher Scientific). HPRT amplification was used as a housekeeping control to standardize the amount of RNA in each sample.

### Protein extraction and transgene detection from the muscles

Proteins were extracted with T-Per Tissue Protein Extraction Reagent (Thermo Fisher Scientific), and the total protein concentration was determined using the Pierce™ BCA Protein Assay Kit (Thermo Fisher Scientific). Equal amounts of protein were used for immunoblotting analysis with PD-L1, PD-L2, and Alkaline Phosphatase antibodies. GAPDH antibody was used as a housekeeping control.

### Histopathology

Tissue samples were collected in 4% formaldehyde fixative (pH 7.4), processed, and embedded in paraffin. Cross-sections (5 µm) of the right gastrocnemius medialis injected with either rAAV6-muSEAP-PD-L1, rAAV6-muSEAP-PD-L2, or rAAV6-muSEAP were stained for CD3, CD4, and CD8 (Cell Signaling Technologies) detection to analyze inflammation state. Ki67 (Cell Signaling Technologies) was used as a proliferation indicator. The benefit of PD-L1/2 depends on PD-1 expression on immune cells, so PD-1 (Cell Signaling Technologies) staining was also conducted. The sections were counterstained with hematoxylin. Sections were imaged with a Nikon H550L microscope, and immune cells were quantified using ImageJ Fiji [31] image processing software.

### Detection of AAV6 and PD-L1/2 IgG antibodies in mouse serum

Serum AAV6 antibody levels were quantified by ELISA. rAAV6 particles were diluted in PBS (pH 7.4) to a final concentration of 1 × 10^8^ vg/μl. The wells of a 96-well Nunc MaxiSorp plate (Thermo Fisher Scientific) were coated with the vector solution and incubated at 4 °C overnight. PBS without rAAV6 was used as a non-coated control. The following day, the plate was washed with PBS and blocked with 5% BSA in PBS by incubation at 37 °C for 2 h. The plate was washed with PBS, and mouse serum, diluted threefold from 1:100 to 1:24300, was added and incubated at 37 °C for 90 min. After washing, mouse IgG peroxidase antibody (Sigma-Aldrich) was added, and the mixture was incubated at 37 °C for 1 h. After washing, tetramethylbenzidine substrate solution (Sigma-Aldrich) was added and incubated for 20 min at room temperature. The reaction was stopped with the stop reagent (Sigma-Aldrich), and absorbances were read at 450 and 650 nm using Varioskan Lux with SkanIt Software. 650 nm readings were subtracted from 450 nm readings to reduce the possible background signal.

PD-L1/2 antibodies were detected in mouse sera using the same method. For antibody capture, plates were coated with recombinant PD-L1 (cat# 9049-B7-100, R&D Systems) or PD-L2 (cat# 9075-PL-100, R&D Systems) proteins in sodium bicarbonate coating buffer (pH 9.5) overnight at room temperature.

### Epitope prediction

Antigenic transgene product epitopes were predicted using the B and T cell epitope prediction tool [32]. B-cell epitopes were predicted using the Kolaskar-Tongaonkar method [33] and T cell epitopes were predicted using TepiTool [34]. For MHC I epitope prediction, 8–11 amino acid-long epitopes were included, and the peptide selection criterion cutoff was set at 1. For MHC II epitope prediction, 15 amino acid-long epitopes were included, and the peptide selection criterion cutoff was set at 10.

### Statistical analysis

Statistical analysis was performed using GraphPad Prism 9.3.0 (San Diego, California, USA). Specific statistical analyses and data presentation are indicated in the figure legends. For normally distributed data, analysis of variance (ANOVA) was used as appropriate. For non-normally distributed data, the Mann-Whitney test, Kruskal-Wallis test, or Wilcoxon test was applied as suitable. Statistical significance is denoted by *(*p* < 0.05), **(*p* < 0.01), ***(*p* < 0.001), and ****(*p* < 0.0001).

## Results

### P2A self-cleavage results in biologically active PD-L1/2 and VEGF-B

**Fig. 1:**
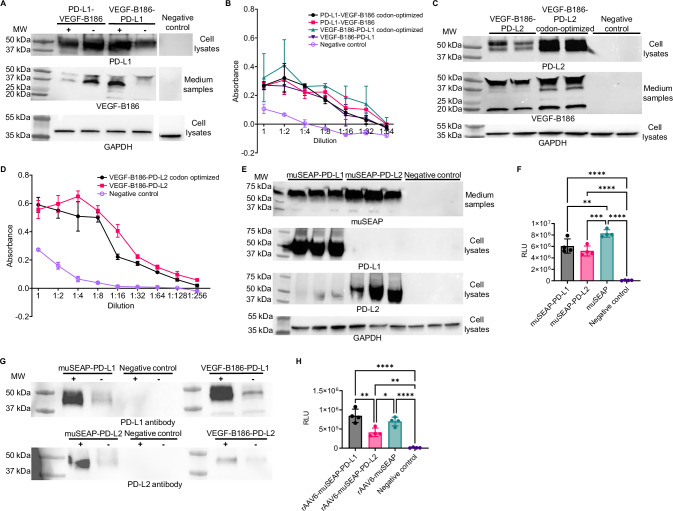
Production and functionality of PD-L1/2, VEGF-B186, and muSEAP. **A** Western blot showing PD-L1 (top) and VEGF-B186 (middle) produced in cell lysates and media of transfected 293T cells, respectively, with (+) and without (–) codon-optimization. **B** BaF3-R1 cell viability after incubation with VEGF-B186-containing media of PD-L1-VEGF-B186 and VEGF-B186-PD-L1-transfected 293T cells. Data are presented as mean (dots) (SD) of two technical replicates from a representative experiment. **C** Western blot showing PD-L2 (top) and VEGF-B186 (middle) produced in cell lysates and media of transfected 293T cells, respectively. **D** BaF3-R1 cell viability after incubation with VEGF-B186-containing media of VEGF-B186-PD-L2-transfected 293T cells. Data are presented as mean (dot) (SD) of two technical replicates from a representative experiment. **E** Western blot showing muSEAP (top) produced into the media and PD-L1 (second row) and PD-L2 (third row) in the cell lysates of transfected 293T cells. **F** Luminescence assay measuring muSEAP secretion into the media of transfected 293T cells. Data are presented as individual values (dot) and mean (column) (SD) of two independent experiments. One-way ANOVA with Tukey’s post hoc test was used for statistical analysis. Mock-treated cells and media were used as negative controls. **G** PD-L1 and PD-L2 detection after pull-down with PD-1-His beads (+). Non-coated beads were used as a control (−). **H**. Luminescence assay measuring muSEAP secretion into the media of rAAV6 transduced 293T cells. Data are presented as individual values (dots) and mean (column) (SD) of two independent experiments. One-way ANOVA with Tukey’s post hoc test was used for statistical analysis. Mock-treated cells and media were used as negative controls, and GAPDH was used as a housekeeping control. MW molecular weight, GAPDH glyceraldehyde 3-phosphate dehydrogenase, RLU relative light unit.

To assess proper cleavage by the P2A peptide, crucial for the functionality of both transgenes, and the effect of transgene codon-optimization on protein production, PD-L1 and VEGF-B186 production were analyzed in 293T cell lysates and media using immunoblotting (Fig. 1A). Successful P2A cleavage was verified in all samples, with correctly sized PD-L1 (approximately 50 kDa) and VEGF-B186 (upper bands approximately 37 and 50 kDa and lower band approximately 20 kDa) proteins detected. Codon-optimized VEGF-B186-PD-L1-transfected cells seemed to produce the highest amounts of both PD-L1 and VEGF-B186. Differences between constructs were particularly evident in media samples when detecting VEGF-B186. The BaF3-VEGFR-1 cell proliferation and survival assay demonstrated that VEGF-B186, expressed from all constructs, was biologically active (Fig. 1B).

PD-L2 constructs were tested with or without VEGF-B186 codon-optimization in the orientation optimized for PD-L1. However, it needs to be noted that the same orientation may not be the most optimal for different proteins. Aligning with the PD-L1 results, cleavage into two proteins was successful, and the codon-optimized VEGF-B186-PD-L2 plasmid produced the highest amounts of VEGF-B186 and PD-L2 (approximately 50 kDa in size) (Fig. 1C). VEGF-B186 was again confirmed to be biologically active (Fig. 1D).

### A secreted marker gene for mouse studies

To further validate the co-expression strategy and enable in vivo monitoring in a proof-of-concept mouse study, we cloned muSEAP combined with PD-L1 or PD-L2. muSEAP can easily be measured in blood samples without sacrificing animals during long-term studies [35]. Immunoblotting of transfected 293T cell lysates and media again confirmed proper P2A activity (Fig. 1E). In addition, a luminescence assay was performed to quantify muSEAP (Fig. 1F). All constructs resulted in biologically active muSEAP secretion into the medium, although the plain muSEAP control plasmid yielded slightly higher levels than the PD-L1/2 co-encoding plasmids.

### Co-produced PD-1 ligands are biologically active

To confirm PD-L1 and PD-L2 binding to their receptor PD-1, we performed a pulldown assay using recombinant PD-1-His protein. Both PD-L1 and PD-L2 bound PD-1 (Fig. 1G). Minor non-specific binding to magnetic beads was observed despite pre-clearing of lysates. To further analyze non-specific binding, we investigated muSEAP and VEGF-B186 in the eluates (Supplementary Fig. 2A). Some VEGF-B186 binding was detected regardless of whether the sample contained PD-1, suggesting that VEGF-B186 non-specifically interacted with the magnetic beads. Additionally, PD-1-His was detected in the eluates (Supplementary Fig. 2B).

### Co-production of functional PD-L1/2 and transgene products using rAAVs

We generated recombinant AAV serotype 6 (rAAV6) with optimized expression cassettes and validated their functionality in 293T cells. Similar to the results seen with plasmids, immunoblotting confirmed the expression of PD-L1 (Supplementary Fig. 3A) and PD-L2 (Supplementary Fig. 3B) in cells and the secretion of VEGF-B186 into the medium (Supplementary Fig. 3C). PD-L1 was clearly detectable in the rAAV6-VEGF-B-PD-L1 and rAAV6-muSEAP-PD-L1-treated lysates, whereas PD-L2 was produced to a lesser degree. VEGF-B186 was detected in all transduced samples.

muSEAP expression was also measured in rAAV6 transduced cell media using a luminescence assay (Fig. 1H). Unlike with plasmids (Fig. 1F), rAAV6-muSEAP-PD-L1 produced slightly more muSEAP than rAAV6-muSEAP, although the difference was statistically insignificant.

### PD-L1 and PD-L2 increase muSEAP expression in pre-immunized mice

**Fig. 2:**
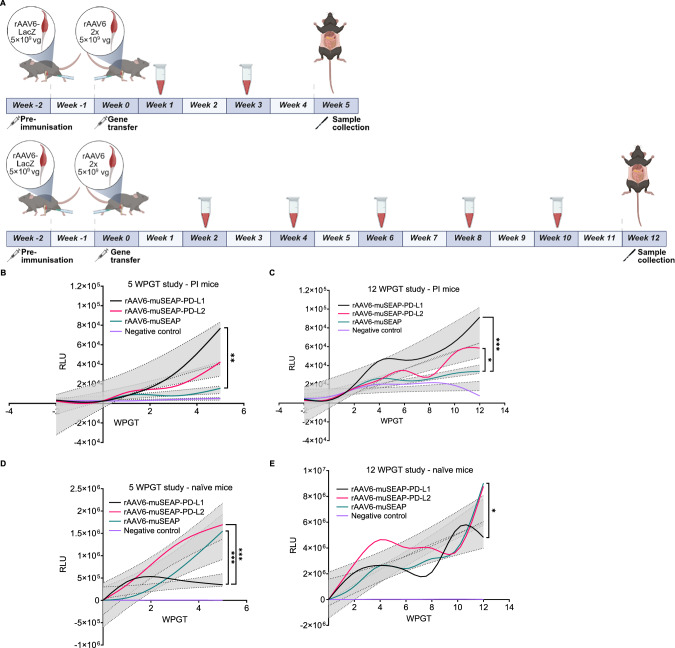
muSEAP detection in mouse serum. **A** A schematic overview of the 5 WPGT (top) and 12 WPGT (bottom) mouse study. Weeks when blood samples were taken are indicated in blue. Figure generated with BioRender. muSEAP measured from the PI mice serum in the 5 WPGT study (**B**) and the 12 WPGT study (**C**), and in naïve mice serum in the 5 WPGT study (**D**) and the 12 WPGT study (**E**). Multiple linear regression was used for statistical analysis. Significances are shown as the effect of treatment as a function of time. Data are presented as means of two independent experiments with LOWESS smoothing and 95% confidence intervals. Mice treated with PBS were used as negative controls. The number of samples are as follows: 5 WPGT study PI mice, rAAV6-muSEAP-PD-L1 *n* = 6, rAAV6-muSEAP-PD-L2 *n* = 4, rAAV6-muSEAP *n* = 4, negative control *n* = 2; 5 WPGT study naïve mice, rAAV6-muSEAP-PD-L1 *n* = 4, rAAV6-muSEAP-PD-L2 *n* = 4, rAAV6-muSEAP *n* = 4, negative control *n* = 2, 12 WPGT study PI mice, rAAV6-muSEAP-PD-L1 *n* = 6, rAAV6-muSEAP-PD-L2 *n* = 6, rAAV6-muSEAP *n* = 6, negative control *n* = 2, 12 WPGT study naïve mice, rAAV6-muSEAP-PD-L1 *n* = 6, rAAV6-muSEAP-PD-L2 *n* = 6, rAAV6-muSEAP *n* = 6, negative control *n* = 2. WPGT weeks post gene transfer, RLU relative light unit.

The effect of PD-L1/2 co-expression on transgene expression and duration was studied in rAAV6-LacZ pre-exposed and naïve mice treated with rAAV6-muSEAP-PD-L1, rAAV6-muSEAP-PD-L2, or a control vector rAAV6-muSEAP. The design of mouse studies is illustrated in Fig. 2A. Two independent experiments were conducted, differing only in duration. The first proof-of-concept study was carried out for 5 weeks post-gene transfer (WPGT), followed by a second study with a longer follow-up period of 12 WPGT.

In both studies, initial muSEAP levels were lower in PI mice compared to naïve mice; however, co-delivery of PD-L1/2 improved muSEAP production over time, with the difference compared to the control group increasing until the final timepoint. rAAV6-muSEAP-PD-L1 consistently showed a statistically significant benefit over the control vector in PI mice (Fig. 2B, C). At 5 WPGT, the increase in muSEAP secretion was 5.9-fold higher, and at 12 WPGT, 3.4-fold higher in the PI PD-L1 co-delivery groups compared to the control group (Supplementary Table 2). At 12 WPGT, the PI PD-L2 group had a 1.6-fold higher increase in muSEAP secretion compared to the plain muSEAP group, which conferred a moderate but statistically significant benefit over the control (Fig. 2C, Supplementary Table 2). The PD-L2 co-delivery group showed a trend of 2.8-fold higher muSEAP production than the control group at 5 WPGT (Fig. 2B, Supplementary Table 2).

In contrast, ligand co-delivery in naïve mice did not result in sustained improvement in muSEAP production (Fig. 2D, E). The initial positive trend associated with PD-L1 declined after two weeks, while PD-L2 co-delivery showed a more sustained, though still transient, positive trend that lasted up to 5 weeks (Fig. 2E).

### PD-L1, PD-L2, and muSEAP were expressed in mouse muscles

**Fig. 3:**
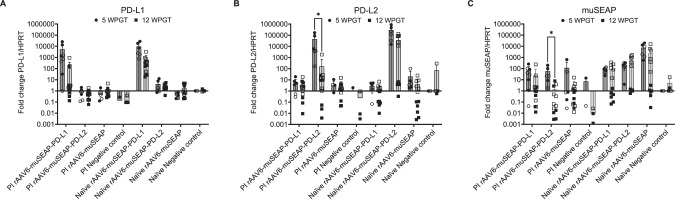
Transgene expression quantified by RT-qPCR in the right gastrocnemius muscle. **A** PD-L1. **B** PD-L2. **C** muSEAP. Gene expression was normalized against a housekeeping gene hypoxanthine phosphoribosyl transferase (HPRT) and is shown as a fold change. Data are presented as individual values (dot = 5 WPGT study, square = 12 WPGT study), with different RNA extraction sites indicated with solid or open symbols, and as median (columns) (IQR) from a representative experiment of three experiments. Mice treated with PBS were used as a negative control. The Mann-Whitney test was used for statistical analysis. The number of samples are as follows: 5 WPGT, PI rAAV6-muSEAP-PD-L1 *n* = 5, PI rAAV6-muSEAP-PD-L2 *n* = 3, PI rAAV6-muSEAP *n* = 3, PI negative control *n* = 1, naïve rAAV6-muSEAP-PD-L1 *n* = 3, naïve rAAV6-muSEAP-PD-L2 *n* = 3, rAAV6-naïve muSEAP *n* = 3, naïve negative control *n* = 1; 12 WPGT PI rAAV6-muSEAP-PD-L1 *n* = 6, PI rAAV6-muSEAP-PD-L2 *n* = 6, PI rAAV6-muSEAP *n* = 6, PI negative control *n* = 2, naïve rAAV6-muSEAP-PD-L1 *n* = 6, naïve rAAV6-muSEAP-PD-L2 *n* = 6, naïve rAAV6-muSEAP *n* = 6, naïve negative control *n* = 2. WPGT weeks post gene transfer.

Transgene expression and protein production were investigated using RT-qPCR and immunoblotting, respectively. RNA was extracted from two randomly selected regions of the treated gastrocnemius muscle. Expression of muSEAP and PD-1 ligands was evident in both studies. PD-L1 (Fig. 3A) and PD-L2 (Fig. 3B) mRNA levels were generally lower in mice sacrificed at the later time point, with a statistically significant reduction observed only for PD-L2 in PI mice. Similarly, muSEAP mRNA levels were lower in PI mice in the longer study, particularly in the PD-L2 group, while expression levels in naïve mice appeared equal (Fig. 3C). These findings reflect successful IM delivery of the vector. However, due to the limited diffusion of AAV from the injection site [36], variability in sampling may have influenced the results.

To assess rAAV6 leakage from muscle, transgene expression was also studied from liver samples. No transgene expression was detected (data not shown).

Despite known challenges in detecting PD-L1 and PD-L2 proteins in tissue [37], we successfully demonstrated their production in transduced muscles (Supplementary Fig. 4 and 5). Consistent with serum muSEAP levels, protein production was more prominent in naïve mouse tissues (Supplementary Fig. 6).

### PD-L1/2 co-expression reduces muscle inflammation in PI mice

To assess T cell infiltration in treated muscle tissue, we stained paraffin-embedded sections with antibodies against CD3, CD4, CD8, Ki67, and PD-1, and quantification analysis was conducted using ImageJ Fiji [31]. Representative stained sections from each study and group are shown in Supplementary Figs. 7–11.

**Fig. 4:**
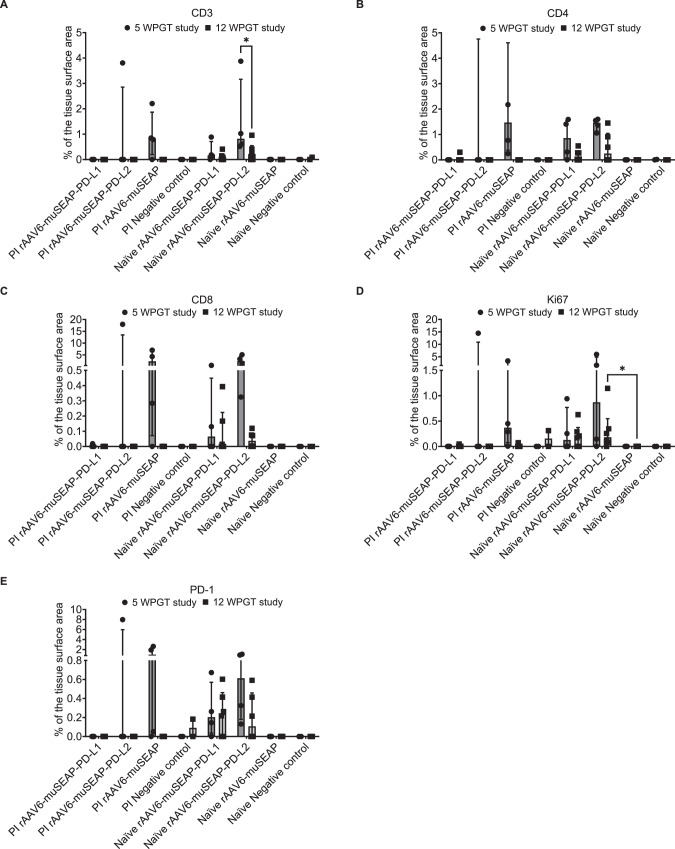
Muscle immune cell profiles at 5 WPGT and 12 WPGT. The prevalence of CD3- (**A**), CD4- (**B**), and CD8-positive cells (**C**), as well as cells expressing Ki67 proliferation marker (**D**) and immune checkpoint molecule PD-1 (**E**), is shown as the stained cell area in the tissue. Data are shown as individual values (dot = 5 WPGT, square = 12 WPGT), and medians are shown as columns (IQR). The Kruskal-Wallis test with Dunn’s post-hoc test was used for statistical analysis between the treatment groups within PI and naïve groups, and the Wilcoxon test was used for statistical analysis between the corresponding PI and naïve groups. The number of samples are as follows: 5 WPGT study, PI rAAV6-muSEAP-PD-L1 *n* = 4, PI rAAV-muSEAP-PD-L2 *n* = 4, PI rAAV6-muSEAP *n* = 4, PI negative control *n* = 2, naïve rAAV6-muSEAP-PD-L1 *n* = 4, naïve rAAV6-muSEAP-PD-L2 *n* = 4, naïve rAAV6-muSEAP *n* = 4, naïve negative control *n* = 2; 12 WPGT study, PI rAAV6-muSEAP-PD-L1 *n* = 6, PI rAAV-muSEAP-PD-L2 *n* = 6, PI rAAV6-muSEAP *n* = 6, PI negative control *n* = 2, naïve rAAV6-muSEAP-PD-L1 *n* = 6, naïve rAAV6-muSEAP-PD-L2 *n* = 6, naïve rAAV6-muSEAP *n* = 6, naïve negative control *n* = 2. WPGT weeks post gene transfer.

In PI mice, PD-L1/2 co-expressing groups had fewer (or undetectable) CD3⁺, CD4⁺, and CD8⁺ T cells than muSEAP-treated mice at 5 WPGT (Fig. 4A–C). In naïve mice, slightly higher numbers of these T cell subsets were detected in the gastrocnemius muscle than in PI mice in PD-L1/2 co-expressing groups 5 WPGT (Fig. 4A–C). However, the overall T cell counts were low, and these differences were not statistically significant, only indicating trends in T cell infiltration.

In the 12 WPGT study, T cell infiltration was predominantly observed in naïve mice co-transduced with PD-1 ligands (Fig. 4A–C). Notably, naïve mice treated with muSEAP-PD-L2 showed elevated levels of CD3⁺ (Fig. 4A) and Ki67⁺ (Fig. 4D) cells compared to the naïve muSEAP group. Overall, inflammation was milder at 12 WPGT than at 5 WPGT, which is consistent with the later sampling time.

In PI mice, the abundance of T cells, Ki67⁺, and PD-1⁺ cells negatively correlated with muSEAP expression, suggesting that reduced inflammation may contribute to improved transgene expression (Supplementary Fig. 12). No such correlation was observed in naïve mice.

### Mouse pre-immunization and gene delivery induce AAV6 antibodies

To verify successful pre-immunization and the production of antibodies following gene transfer, total AAV6 antibody levels in serum samples from PI and naïve mice were measured by ELISA. Statistical analysis was conducted with dilution factor normalized values (data not shown).

**Fig. 5:**
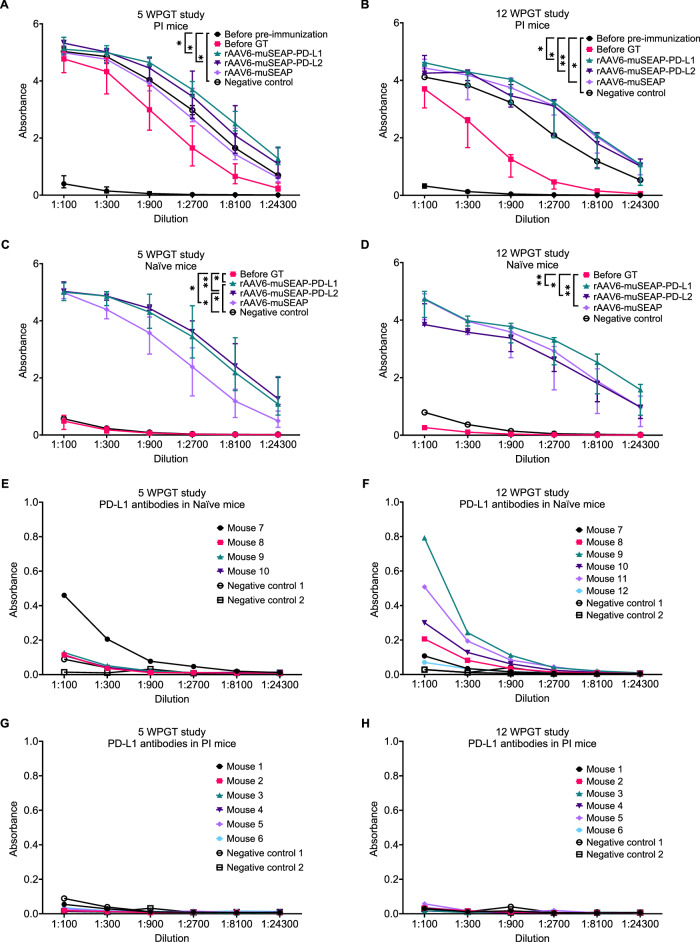
AAV6 and hPD-L1 antibodies in mouse serum. AAV6 antibodies in PI mice in the 5 WPGT study (**A**) and 12 WPGT study (**B**), and in naïve mice in the 5 WPGT study (**C**) and 12 WPGT study (**D**). Data are presented as median (dots) (range) from representative experiments of two (5 WPGT study) and one (12 WPGT study) experiments. The Kruskal-Wallis test with Dunn’s post hoc test was used for statistical analysis. rAAV6-muSEAP-PD-L1, rAAV6-muSEAP-PD-L2, and rAAV6-muSEAP termination serum samples were used in both studies. Mice treated with PBS were used as negative controls. The number of samples are as follows: 5 WPGT study PI mice, before pre-immunization *n* = 2, before GT n = 2, rAAV6-muSEAP-PD-L1 *n* = 3, rAAV6-muSEAP-PD-L2 *n* = 3, rAAV6-muSEAP *n* = 3, negative control *n* = 1; 5 WPGT study naïve mice, before GT *n* = 2, rAAV6-muSEAP-PD-L1 *n* = 3, rAAV6-muSEAP-PD-L2 *n* = 3, rAAV6-muSEAP *n* = 3, negative control *n* = 1; 12 WPGT study PI mice, before pre-immunization *n* = 2, before GT *n* = 3, rAAV6-muSEAP-PD-L1 *n* = 3, rAAV6-muSEAP-PD-L2 *n* = 3, rAAV6-muSEAP *n* = 3, negative control *n* = 1; 12 WPGT study naïve mice, Before GT *n* = 3, rAAV6-muSEAP-PD-L1 n = 3, rAAV6-muSEAP-PD-L2 *n* = 3, rAAV6-muSEAP *n* = 3, negative control *n* = 1. PD-L1 antibodies in mouse serum of naïve mice from the 5 WPGT study (**E**) and 12 WPGT study (**F**), and PI mice from the 5 WPGT study (**G**) and the 12 WPGT study (**H**). rAAV6-muSEAP-PD-L2 (Negative control 1) and PBS (Negative control 2) treated mice were used as negative controls. Data are representative of two independent experiments. GT gene transfer, WPGT weeks post gene transfer.

Antibody formation was detected after pre-immunization and gene transfer, and the immune response persisted throughout the study period (Fig. 5A–D). In PI mice, antibodies were already present following pre-immunization, and they continued to rise after gene transfer (Fig. 5A, B). Most vector-treated groups showed a significant increase in antibody levels post gene transfer, except for the rAAV6-muSEAP-treated PI mice in the 5 WPGT study, where the increase was insignificant (Fig. 5A).

No previous exposure to AAV6 was detected, nor were there increases in antibodies in the negative control groups of naïve mice (Fig. 5C, D).

### Human PD-L1 elicits an antibody response in mice

To determine whether the human PD-1 ligands triggered an immune response in mice, the presence of antibodies against the human ligands in serum samples was measured at the study endpoint. Human PD-L1 antibodies were detected exclusively in naïve mice (Fig. 5E, F). The prevalence of PD-L1 antibodies was higher in animals from the 12 WPGT study compared to the 5 WPGT mice. In contrast, no antibodies against PD-L2 were detected in any of the 12 WPGT study animals (Supplementary Fig. 13A and B), implying a lower immunogenic profile for PD-L2 under the tested conditions.

To better understand the observed differences in immunogenicity between the ligands, we performed a basic in silico prediction of antigenic epitopes for PD-L1, PD-L2, muSEAP, and their theoretical fusion products. This was carried out using the widely adopted algorithms for B cell (Kolaskar-Tongaonkar method [33]) and MHC-binding T cell (TepiTool [34]).

The prediction revealed fewer possible B cell epitopes in muSEAP-PD-L2 and plain PD-L2 than their PD-L1 counterparts (Table 1), though the differences were modest. Similarly, PD-L2 had fewer predicted MHC class I epitopes than PD-L1, while, interestingly, it had slightly more predicted MHC class II epitopes. However, the differences in MHC epitopes are even smaller than those in B cell epitopes, differing only by one epitope. While it is important to note that these predictions do not account for epitope quality or immunodominance, and deeper antigenic profiling is warranted, the results are consistent with the antibody response data.

**Table 1:** Predicted B and T cell epitopes for muSEAP, PD-L1, PD-L2, and P2A uncleaved versions of the transgene products.

	B cell epitopes	MHC I epitopes	MHC II epitopes
**muSEAP-PD-L1**	30	106	11
**muSEAP-PD-L2**	25	105	12
**muSEAP**	19	66	9
**PD-L1**	11	39	2
**PD-L2**	8	38	3

## Discussion

Immune responses limit the clinical use of rAAV as a gene therapy vector and restrict vector re-administration. This particularly concerns therapeutic targets such as non-immune-privileged skeletal muscle, which often require high vector doses. Although tissues, such as the eye and brain, are immunoprotected to a certain degree, they are not entirely immune-privileged either. Current clinical strategies to manage immune responses include excluding seropositive individuals and the use of immunosuppressants [23]. Despite significant progress in patient care, vector-related toxicities, some resulting in fatal outcomes, remain a concern, as evidenced by the clinical trials of Duchenne muscular dystrophy, spinal muscular atrophy, and X-linked myotubular myopathy [38]. Thus, although proven generally safe, there is a pressing need to improve the safety and efficacy of AAV-mediated gene therapy.

PD-1 and its ligands constitute a key immune checkpoint pathway that drives tolerance to autoantigens by controlling T-cell activation and proliferation [39–41]. While PD-L1 overexpression in tumors can result in unwanted T-cell exhaustion and apoptosis, enabling tumor immune escape [26, 27], it also has therapeutic potential in gene therapy by promoting immune tolerance. Although PD-1 is conserved across species [42, 43], there are still substantial differences in its function between humans and mice. The affinity of PD-1 to its ligands varies [44]; human PD-L2 has a higher affinity to human PD-1 than human PD-L1 (0.19 and 6.39 µM, respectively), whereas human PD-L1 has a higher affinity for mouse PD-1 than PD-L2 (4.02 and 10.69 µM, respectively). Furthermore, PD-1’s immunosuppressive activity is weaker in mice than in other mammals [45], suggesting that the immunomodulatory effects observed in mice may have greater therapeutic potential in humans.

To mitigate vector-induced inflammatory responses in hopes of improving the therapeutic outcome, we created rAAV6 vectors which co-delivered PD-1 ligands PD-L1 or PD-L2 with a transgene. This strategy led to a stronger and more sustained gene expression in mouse muscles compared to plain transgene delivery. Our findings contradict those of Adriouch et al. [46], who previously reported limited success using a seemingly similar approach but using separate AAV8 vectors for ligand and transgene delivery in AAV-naïve mice. Their approach likely suffered from insufficient co-transduction of target cells, which is essential for a successful outcome. In contrast, our single-vector strategy ensured co-expression of PD-1 ligands with the transgene.

While preparing this manuscript, McMurphy et al. [47] reported findings similar to ours, using co-delivery of mouse PD-L1 with a luciferase-encoding AAV8 vector. However, they focused solely on PD-L1, used tissue-specific promoters, and conducted the study exclusively in AAV-naïve animals. In contrast to their findings, we did not observe favorable results in naïve mice, likely due to the anti-hPD-L1 response, as discussed below. Additionally, the use of a strong CMV promoter, which drives higher transgene expression compared to the tissue-specific promoters used by McMurphy et al., may also account for the observed differences. Higher protein production increases the likelihood of triggering immune responses [48]. Further studies are warranted to better understand the differences in the results.

We observed a clear benefit of PD-L1/2 co-delivery only in PI mice. The muSEAP-PD-L1-treated mice showed a significant improvement in serum muSEAP levels over time compared to the control group, while the muSEAP-PD-L2 animals exhibited significantly higher muSEAP production only in the 12 WPGT study. In contrast, naïve mice showed a reversed trend; muSEAP-PD-L2 mice displayed higher serum muSEAP levels than muSEAP-PD-L1 animals, but neither group demonstrated any statistically significant advantage over the control group. These differences may be due to variations in ligand antigenicity, ligand expression levels, or the elevated anti-AAV response in the PI mice. The co-delivery strategy likely provided an advantage in PI mice, whose immune systems had been previously primed against the vector, while the human-derived ligands served as immunologically novel antigens. This may have allowed them a longer window to exert immunomodulatory effects before being targeted by the host immune response, while naïve mice had both immunogens introduced simultaneously. This is also consistent with the greater infiltration of immune cells in the treated muscle and humoral anti-ligand response in naïve mice. Notably, only PD-L1 induced a detectable antibody response exclusively in naïve mice, despite the relatively similar protein-sequence homology between human and murine PD-L1 (69%) and PD-L2 (70%) [39]. However, the epitope prediction analysis revealed slightly more B-cell epitopes in PD-L1 than in PD-L2 (11 and 8, respectively). Interestingly, in PI mice, PD-L1 was more effective than PD-L2, possibly due to a lack of a hPD-L1 humoral response and its higher affinity for mouse PD-1. Supporting the immunomodulatory role of PD-1 ligands, immune cell infiltration was generally lower in the PI ligand groups.

Our study was designed to reflect a clinically relevant dose and situation, where most patients are pre-exposed to AAVs. We confirmed successful AAV pre-immunization by measuring AAV6 antibodies, which remained stable after gene transfer (AAV re-dosing). Since only total antibody levels were assessed, differences in the proportions of binding and neutralizing antibodies between PI and naïve mice may have influenced the observed variation in transduction efficacy. However, some previous studies have suggested a correlation between anti-AAV IgG levels and neutralizing antibody levels [11]. Importantly, PD-1 ligands were not expected to affect the anti-AAV levels, nor was such a phenomenon observed.

A limitation of the co-delivery strategy is the small, about 5 Kbp, packaging capacity of rAAV, which is not the case with other vectors, such as adenovirus, lentivirus, or herpes simplex virus. Fortunately, PD-L1/2 encoding genes are relatively small (873 bp and 822 bp, respectively) and, as we show in this study, can be separated from the therapeutic protein with a short (66 bp and 22 aa) self-cleaving P2A peptide without compromising protein functionality. Indeed, the presented new concept is universally applicable to potentially reduce inflammatory responses against the vector and transgene.

In conclusion, we have demonstrated the potential of PD-1 ligands to enhance transgene expression in animals pre-exposed to AAV, when co-delivered with a transgene in a single vector. Using an AAV6 vector encoding muSEAP, we showed enhanced and prolonged transgene expression in the presence of an active mouse immune system. However, this strategy is also applicable to other vectors and could be combined with additional immunosuppressive agents or genetic components, such as corticosteroids, calcineurin inhibitors [23], or TLR-9 inhibitor element [24], to further improve the outcomes.

## Supplementary information

Supplementary information

Supplementary Table 2

## Data Availability

All data generated in this study are included in this published article and the supplemental section of the manuscript. Raw data will be made available upon reasonable request.
