# Supplementary material for: The co-delivery of Programmed Death 1 ligands enhances and prolongs rAAV-mediated gene expression in pre-immunized mice
Source: Gene Ther. 2026 Jan 9;33(2):127–37. doi: 10.1038/s41434-025-00588-9 (PMC13056579; doi:10.1038/s41434-025-00588-9)
Supplement: Supplementary file 1 — Supplementary information [file 41434_2025_588_MOESM1_ESM.pdf]

Supplementary information

Supplementary Table 1. Primer information.

codon optimized PD-L1 primers	Forward	GGT GGA CCC TGT GAC ATC TG
	Reverse	GCT GCT GGT CCA GAT CAC TT
codon optimized PD-L2 primers	Forward	GTT CAC AGT GAC CGT GCC TA
	Reverse	GGC TTG TGT CGT TTT CCA CC
muSEAP primers	Forward	GTA GCC TAT CGT GCC CTG AC
	Reverse	TGC CAT CCT CAG CCT TGA AG
Mouse HPRT primers	Forward	GAT CAG TCA ACG GGG GAC AT
	Reverse	GGG GCT GTA CTG CTT AAC CA

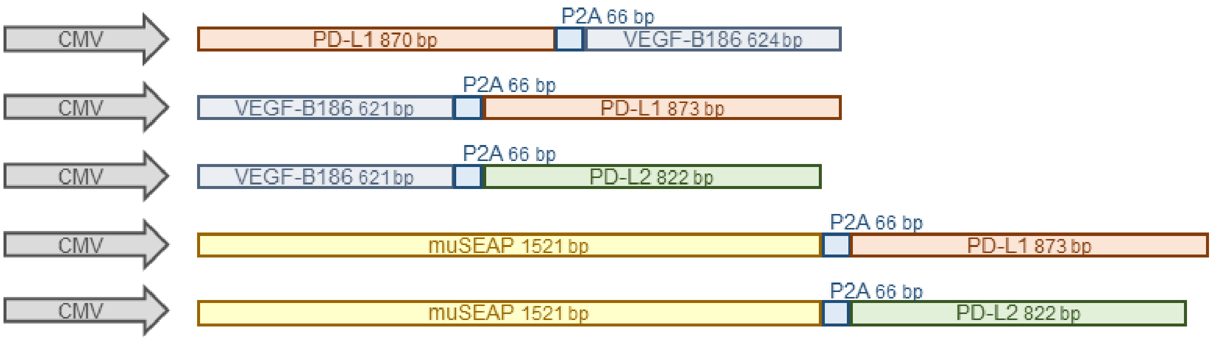

Supplementary Figure 1. Schematic representation of tested constructs.

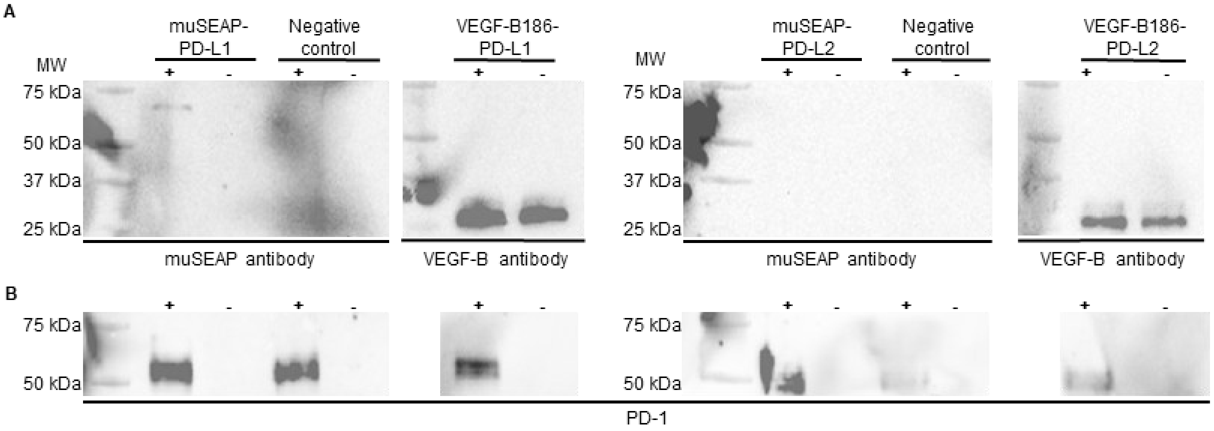

Supplementary Figure 2. Western blot analysis of transfected 293T cell lysates following pull-down with recombinant PD-1-His protein (+) or control magnetic beads (−). A muSEAP and VEGF-B186 detection after pull-down with PD-1-His beads. **C** PD-1 detection after pull-down with PD-1-His beads. The mock-treated cell lysate was used as a negative control. MW = molecular weight.

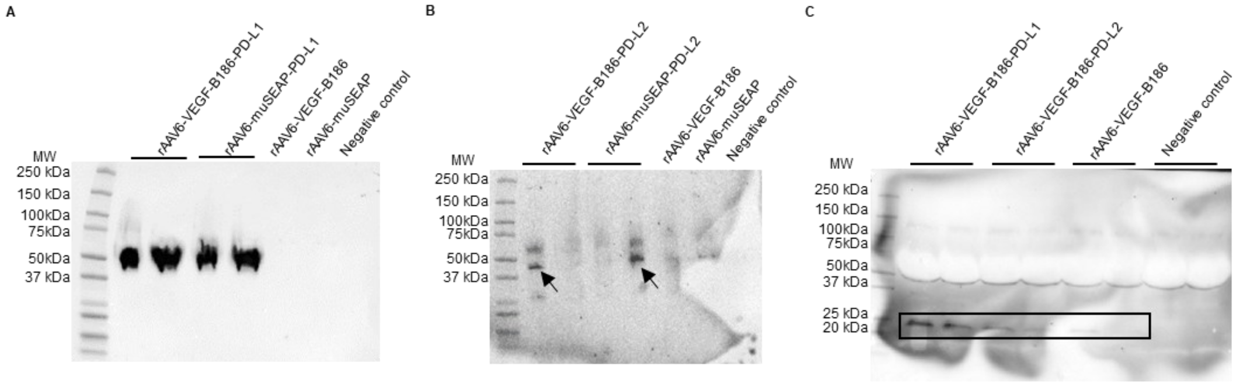

Supplementary Figure 3. Protein production in rAAV6-VEGF-B186-PD-L1 and rAAV6-VEGF-B186-PD-L2 transduced cells. A PD-L1 **B** PD-L2 **C** VEGF-B186. MW = molecular weight.

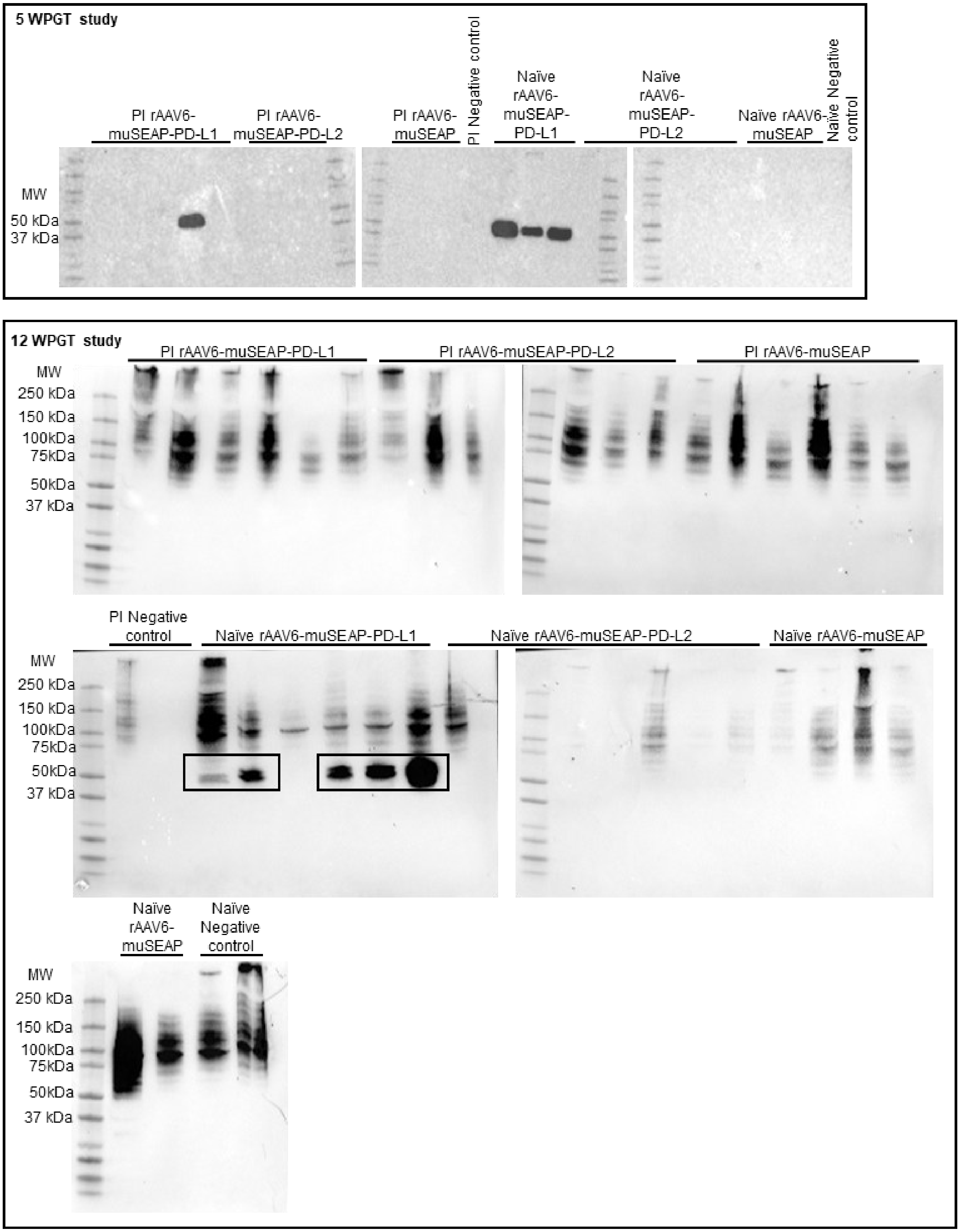

Supplementary Figure 4. PD-L1 production in the right gastrocnemius muscle. MW = molecular weight.

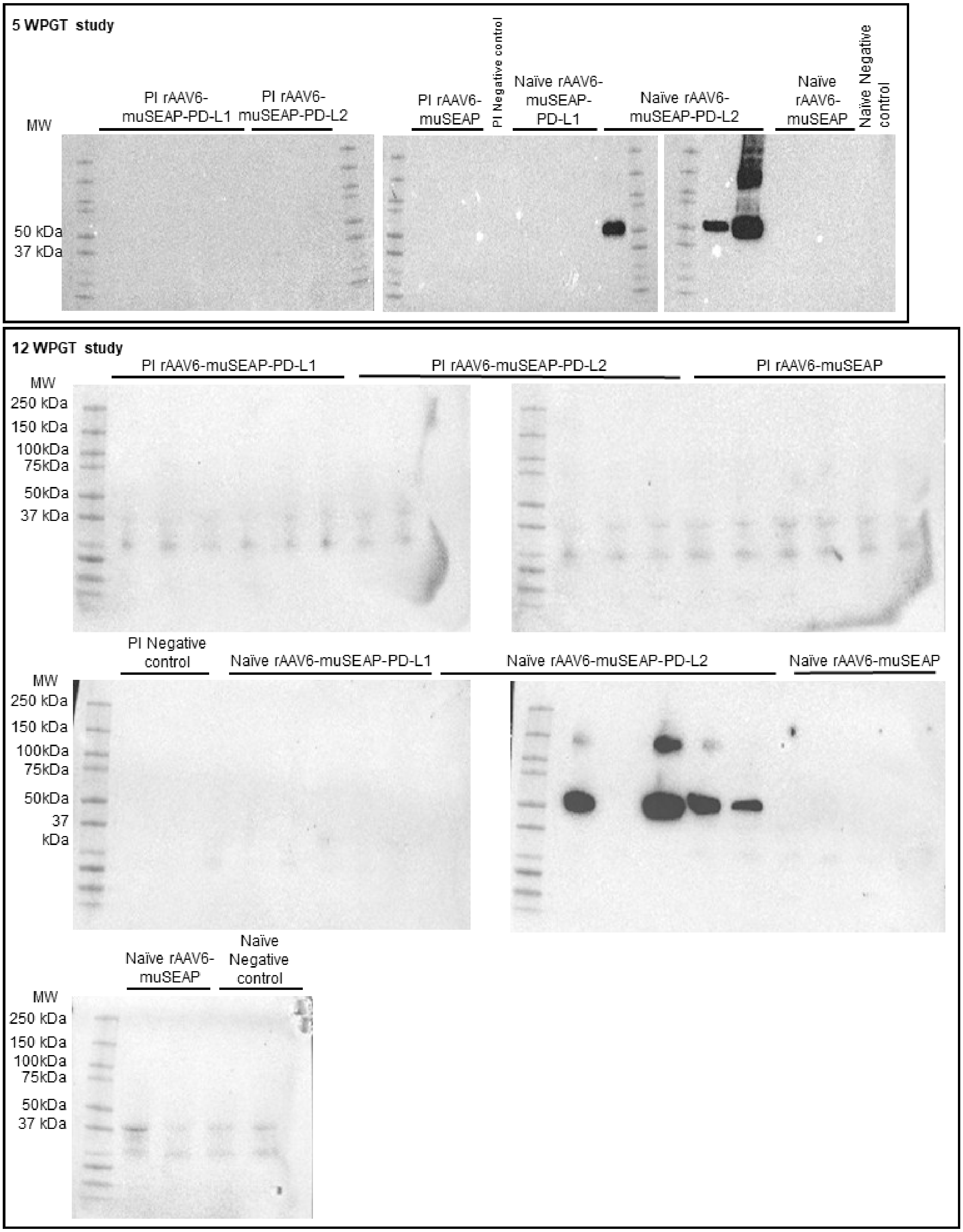

Supplementary Figure 5. PD-L2 production in the right gastrocnemius muscle. MW = molecular weight.

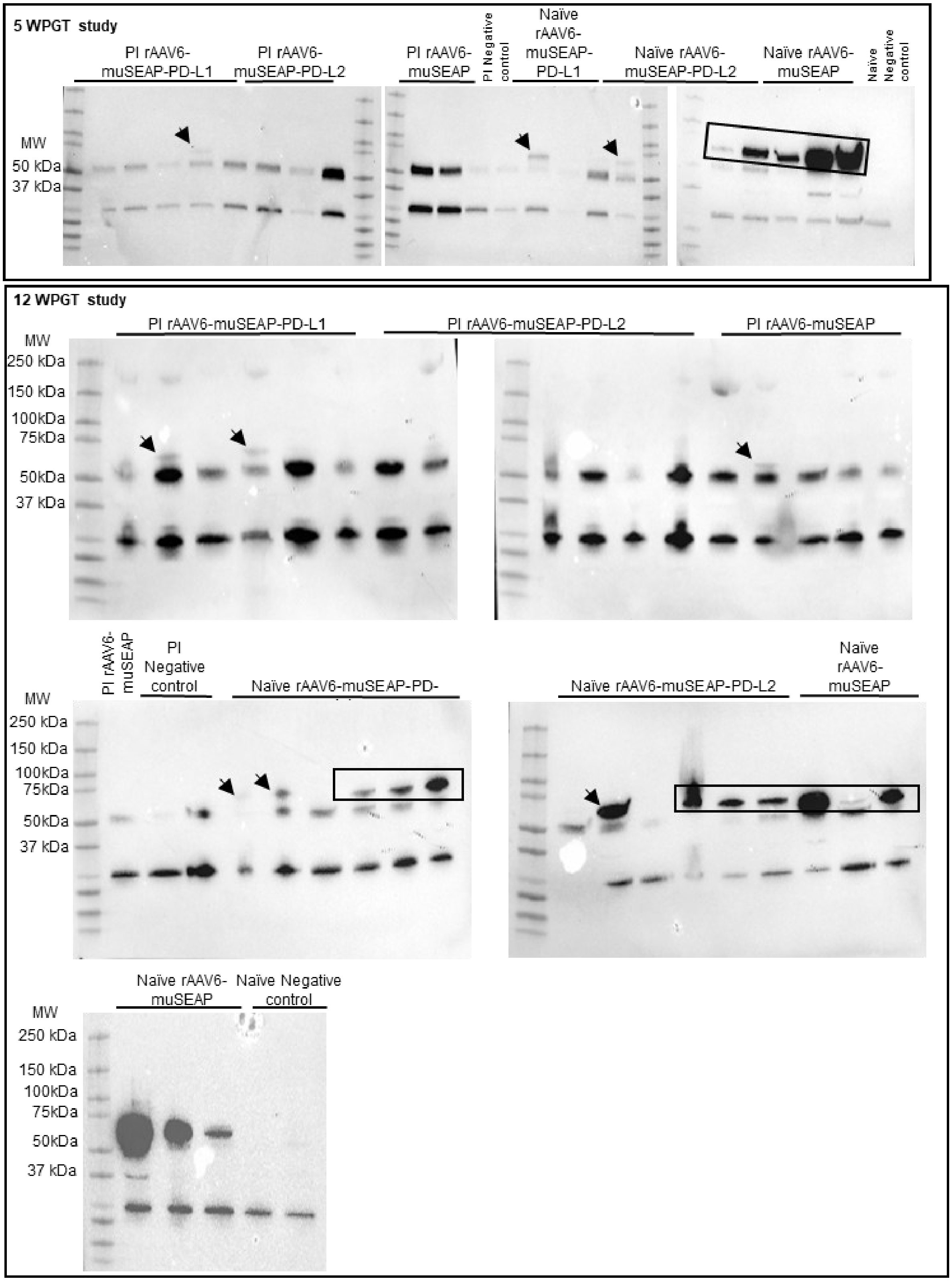

Supplementary Figure 6. muSEAP production in the right gastrocnemius muscle. Mouse secondary antibody was used; thus, IgG light (25 kDa) and heavy (50 kDa) chains are also detected. Correct bands are indicated with arrows and rectangles. MW = molecular weight.

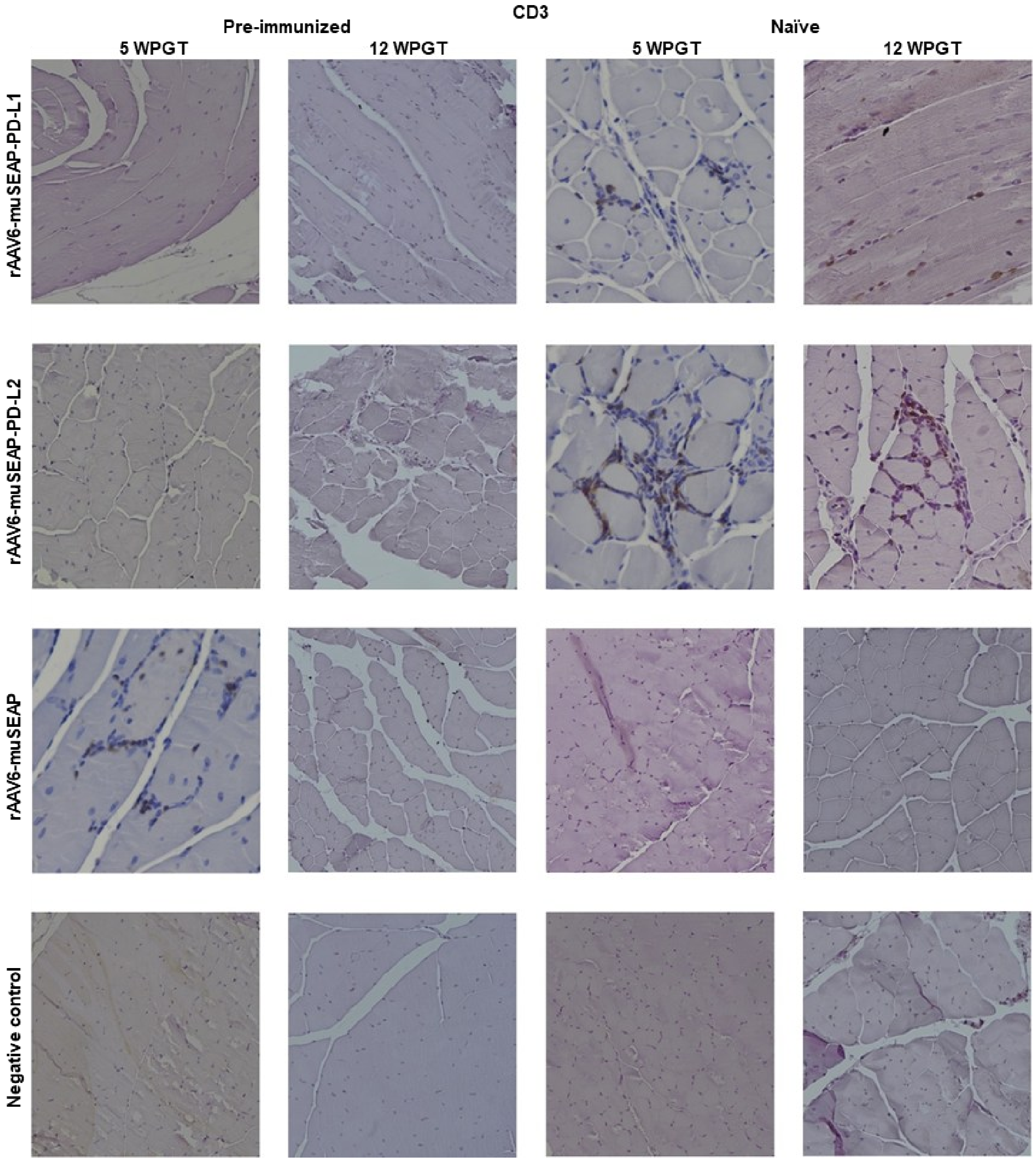

Supplementary Figure 7. CD3 staining of the right gastrocnemius medialis. One representative section is shown per group.

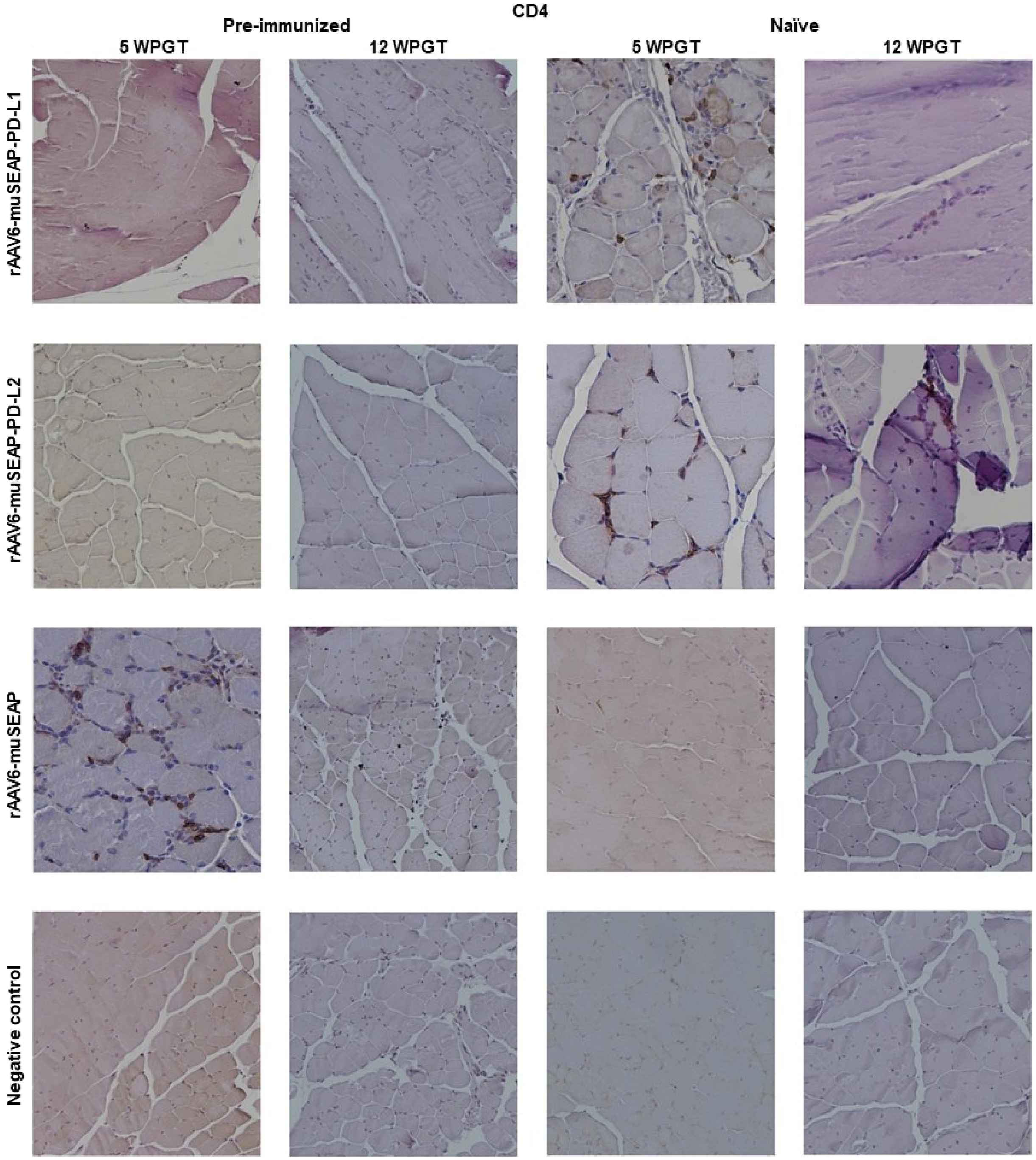

Supplementary Figure 8. CD4 staining of the right gastrocnemius medialis. One representative section is shown per group.

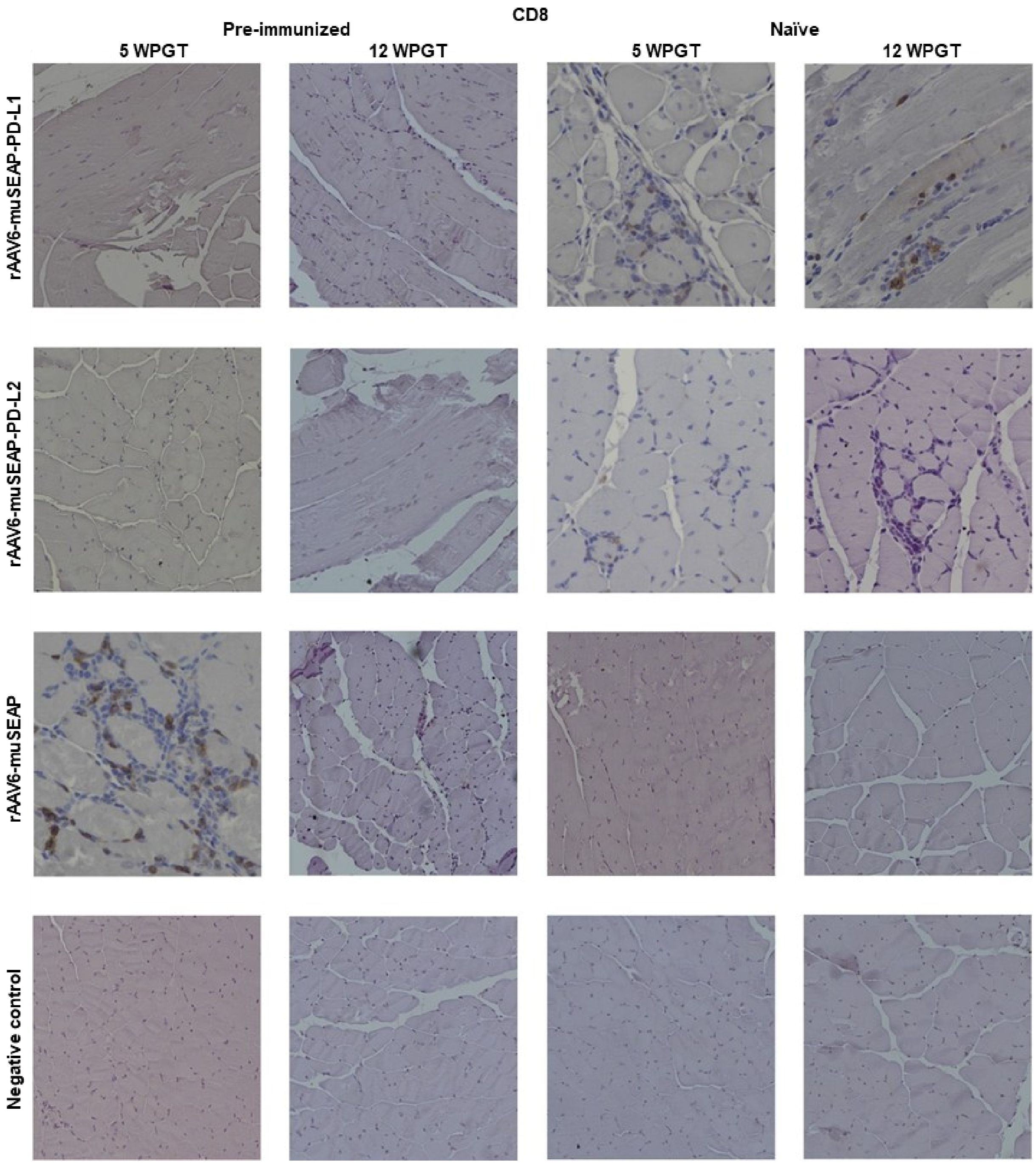

Supplementary Figure 9. CD8 staining of the right gastrocnemius medialis. One representative section is shown per group.

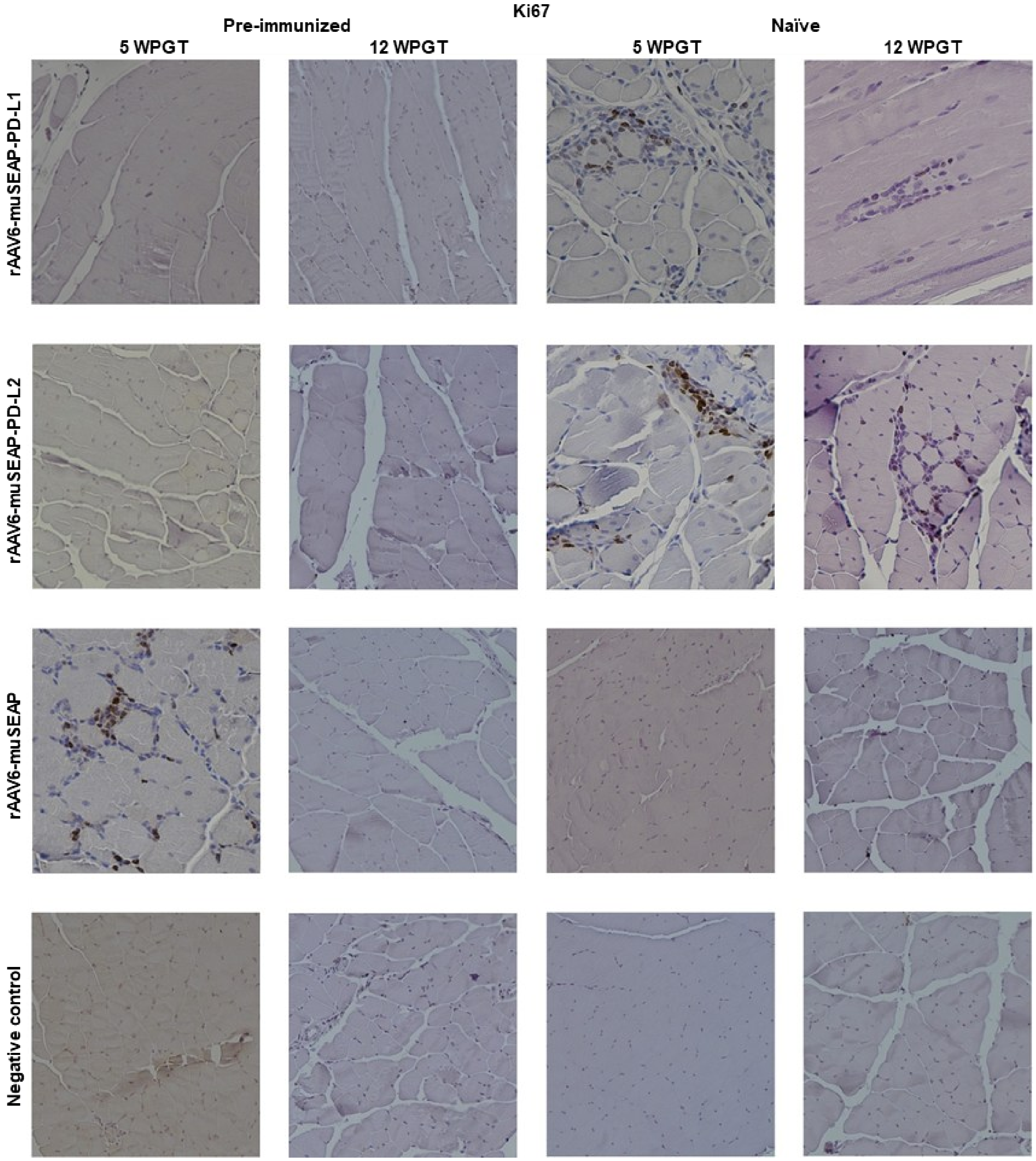

Supplementary Figure 10. Ki67 staining of the right gastrocnemius medialis. One representative section is shown per group.

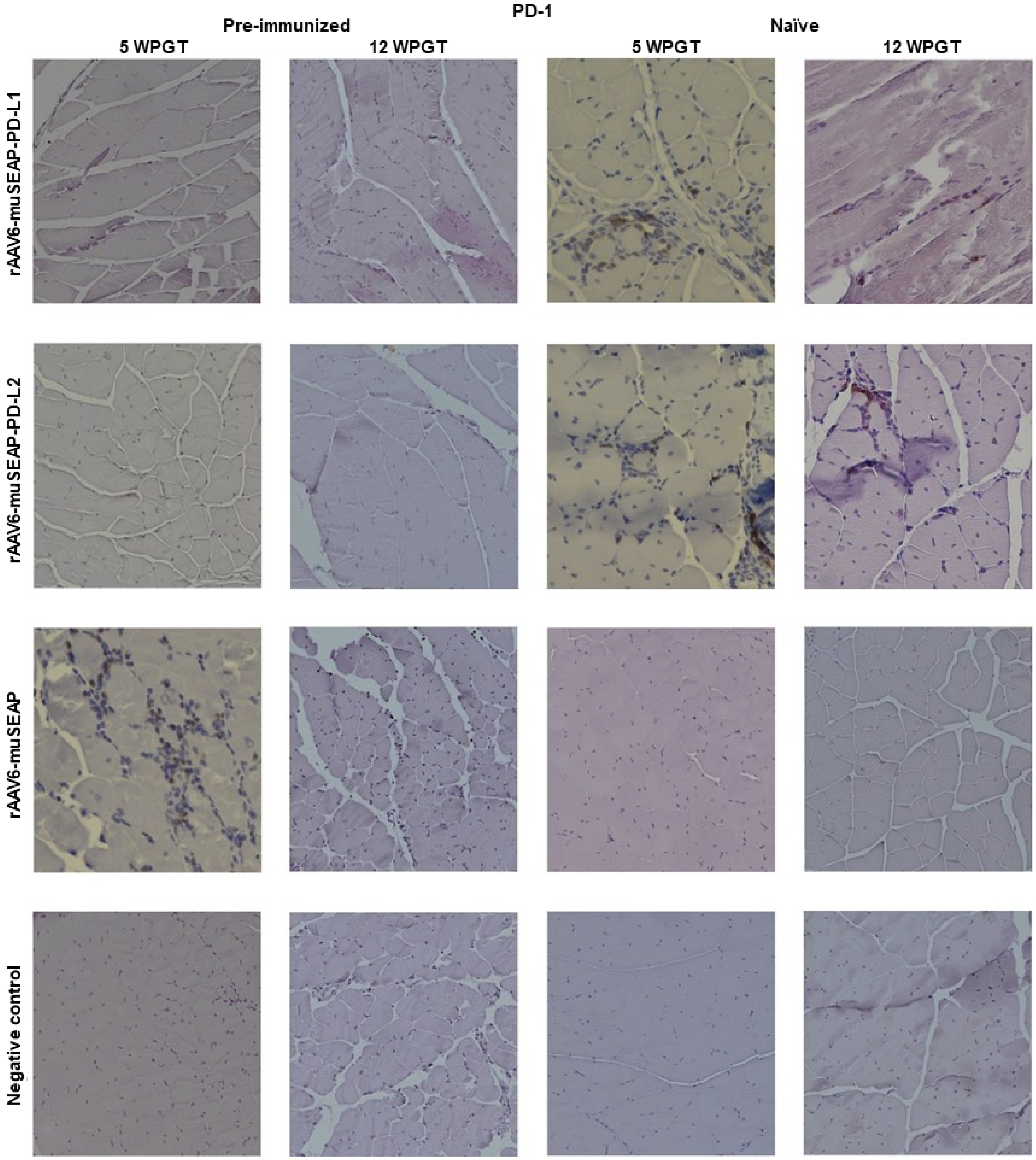

Supplementary Figure 11. PD-1 staining of the right gastrocnemius medialis. One representative section is shown per group.

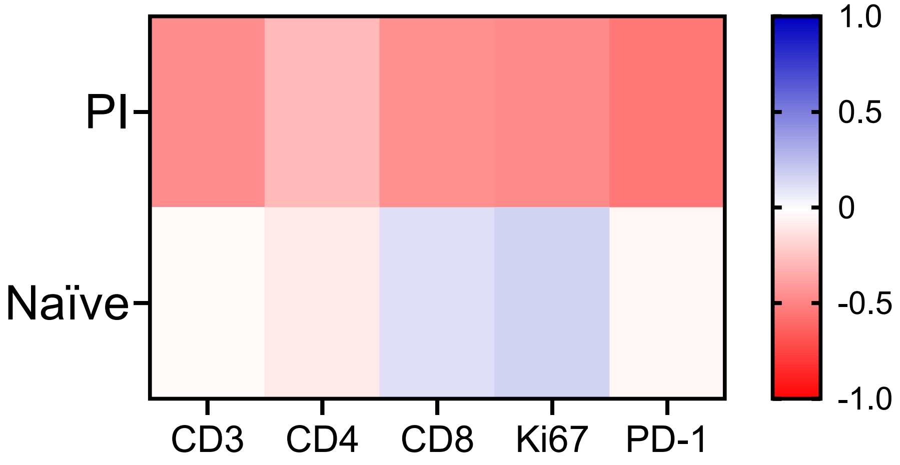

Supplementary Figure 12. Correlation between T cell markers (CD3, CD4, and CD8), proliferation marker Ki67, and immune checkpoint PD-1 and luminescence (muSEAP) levels in PI and naïve mice.

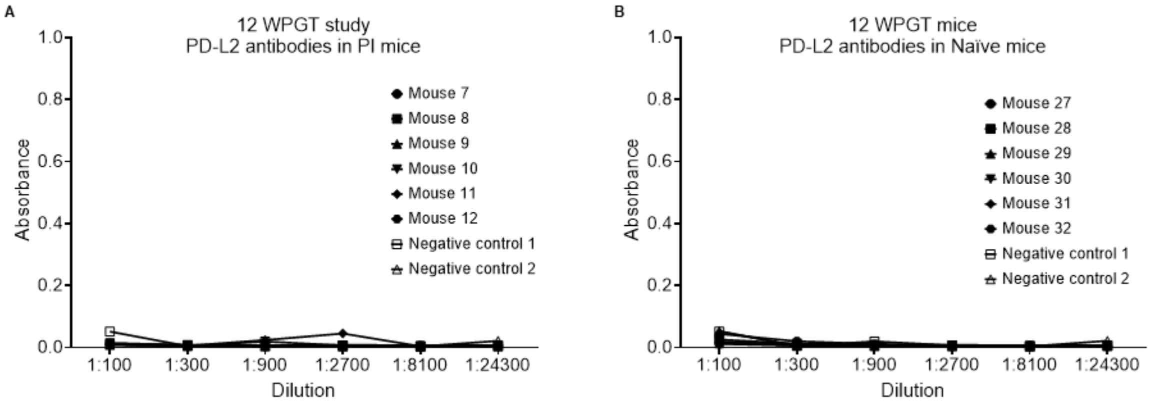

Supplementary Figure 13. Human PD-L2 antibodies in mouse serum. A PD-L2 antibodies in the 12 WPGT study PI mice. **B** PD-L2 antibodies in the 12 WPGT study naïve mice**.** Individual mice are shown as dots. rAAV6-muSEAP-PD-L1 (Negative control 1) and PBS (Negative control 2) treated mice were used as negative controls.
